# Gallbladder Cancer in the 21st Century

**DOI:** 10.1155/2015/967472

**Published:** 2015-09-01

**Authors:** Rani Kanthan, Jenna-Lynn Senger, Shahid Ahmed, Selliah Chandra Kanthan

**Affiliations:** ^1^Department of Pathology & Laboratory Medicine, University of Saskatchewan, Saskatoon, SK, Canada S7N 0W8; ^2^Department of Surgery, University of Alberta, Edmonton, AB, Canada T6G 2B7; ^3^Division of Medical Oncology, Division of Medical Oncology, University of Saskatchewan, Saskatoon, SK, Canada S7N 0W8; ^4^Department of Surgery, University of Saskatchewan, Saskatoon, SK, Canada S7N 0W8

## Abstract

Gallbladder cancer (GBC) is an uncommon disease in the majority of the world despite being the most common and aggressive malignancy of the biliary tree. Early diagnosis is essential for improved prognosis; however, indolent and nonspecific clinical presentations with a paucity of pathognomonic/predictive radiological features often preclude accurate identification of GBC at an early stage. As such, GBC remains a highly lethal disease, with only 10% of all patients presenting at a stage amenable to surgical resection. Among this select population, continued improvements in survival during the 21st century are attributable to aggressive radical surgery with improved surgical techniques. This paper reviews the current available literature of the 21st century on PubMed and Medline to provide a detailed summary of the epidemiology and risk factors, pathogenesis, clinical presentation, radiology, pathology, management, and prognosis of GBC.

## 1. Introduction

Gallbladder cancer is the most common malignant tumour of the biliary tract worldwide [1]. It is also the most aggressive cancer of the biliary tract with the shortest median survival from the time of diagnosis [2]. This poor prognosis is due, in part, to an aggressive biologic behavior and a lack of sensitive screening tests for early detection resulting in delayed diagnosis at advanced stage [3]. The only chance for a complete cure is by surgical resection; however, at initial presentation, only 10% of patients are candidates for surgery with a curative intent [2]. Even among those suitable for resection, the anatomical complexity of the portobiliary hepatic system, the morbidity/mortality associated with liver resection, and the risks of tumoural spread second to tumour manipulation portend a high mortality rate [4]. Additionally, among those that do undergo surgical resection, recurrence rates remain high [2].

This paper adds to the body of existing literature in gallbladder carcinomas to enhance awareness of this uncommon but otherwise potentially curable disease. In this paper, we review salient features of the epidemiology and risk factors, pathogenesis, clinical presentations, imaging findings, pathology, and prognosis of gallbladder cancer with special emphasis on advances in the management of gallbladder cancer through evidence-based reviews published in the 21st century (2000–present).

## 2. Methodology

A systematic review of the published medical literature using PubMed and Medline was carried out using the search terms “gallbladder” AND “cancer [OR] carcinoma” with a special emphasis on review articles. Secondary references obtained from these publications were identified by a manual search and reviewed as relevant. Case reports except for rare pathological entities were predominantly excluded. Manuscripts focusing on gallbladder cancer were included in this review, while those dedicated exclusively to biliary tract malignancies were excluded. Selected relevant abstracts from key oncology meetings (American Society of Clinical Oncology, European Cancer Congress, Gastrointestinal Cancer Symposium, World Congress on Gastrointestinal Cancer, and Society of Surgical Oncology meeting) have also been reviewed. We have predominately limited our search to publications since 2000 to review concepts of gallbladder cancer in the 21st century.

## 3. Epidemiology and Risk Factors

Estimates by the American Cancer Society suggest 10,910 new cases of GBC will be diagnosed in the United States in 2015, with 3,700 deaths [5]. Gallbladder cancer is three times more common in females than in males [1, 6]. Among women, higher gravidity and parity increase the risk of developing this cancer [7]. The incidence of gallbladder cancer increases with age [1]. Within the United States, GBC is more prevalent among Mexican Americans and Native Americans, two populations who also have higher rates of gallstones. The average age at diagnosis is 72, with more than two out of three people with GBC over the age of 65 years [5].

The pathogenesis of gallbladder cancer is likely multifactorial, with no single causative factor being identified.

Risk factors for gallbladder cancer can be divided into four broad categories as annotated in the following list including (1) patient demographics, (2) gallbladder abnormalities, (3) patient exposures, and (4) infections [1, 8, 9].

Risk factors for the development of gallbladder cancer are listed as follows:

1. Demographic factors:
  - advanced age,
  - female gender,
  - obesity,
  - geography: South American, Indian, Pakistani, Japanese, and Korean,
  - ethnicity: Caucasians, Southwestern Native American, Mexican, and American,
  - genetic predisposition.
2. Gallbladder pathologies/abnormalities:
  - cholelithiasis,
  - porcelain gallbladder,
  - gallbladder polyps,
  - congenital biliary cysts,
  - pancreaticobiliary maljunction anomalies.
3. Exposures:
  - heavy metals,
  - medications: methyldopa, OCP, isoniazid, and estrogen,
  - smoking.
4. Infections:
  - * Salmonella*,
  - * Helicobacter*.

### 3.1. Demographic Factors

A striking geographical variability is observed in the prevalence of gallbladder carcinoma worldwide. Regions reporting a high incidence of gallbladder cancer include Delhi, India (21.5/100,000), La Paz, Bolivia (15.5/100,000), South Karachi, Pakistan (13.8/100,000), and Quito, Ecuador (12.9/100,000) [1]. High rates are reported in Chile (27/100,000), Poland (14/100,000), Japan (7/100,000), and Israel (5/100,000) [6]. Northern India, Korea, Japan, and central/eastern Europe including Slovakia, Czech Republic, and Slovenia have also reported a higher prevalence than the worldwide average [1]. By contrast, gallbladder cancer is rare in the western world (USA, UK, Canada, Australia, and New Zealand) with incidence rates of 0.4–0.8 in men and 0.6–1.4 in women per 100,000 [10]. In keeping with this, a retrospective review of the International Agency for Research on Cancer identified increasing rates of male gallbladder cancer mortality only in Iceland, Costa Rica, and Korea with declining rates in all other countries studied [11]. This geographic variability is most likely attributable to differences in environmental exposures and a regional intrinsic predisposition to carcinogenesis [7]. This genetic predisposition is proposed to originate from population migration patterns in Central Asia/Himalayas through Bering Strait during the last glacial era [12]. However, though such patterns of prevalence are observed, there is no true unifying factor that explains this unusual geographic distribution. Alternatively, this variability may be due to dietary factors, with diets high in calories, carbohydrates, red meats, oils, and red chili peppers conferring a higher risk. Intake of green leafy vegetables and fruits may be protective [9]. In keeping with this observation, obesity is a well-recognized risk factor for the development of gallbladder cancer. For each 5-point increase in BMI, the relative risk of developing gallbladder cancer increases by 1.59 for women and 1.09 for men [7, 13].

### 3.2. Gallbladder Pathologies

#### 3.2.1. Cholelithiasis

The most important risk factor for the development of gallbladder cancer is gallstones, with an 8.3x higher risk than the general population [6]. Among patients with gallbladder cancer, 70–90% have a history of cholelithiasis [8, 9]. Larger stones portend a greater risk, with stones >3 cm being 9.2–10.1 times greater than stones <1 cm [1]. This increased risk is most likely attributable to greater local epithelial irritation. Gallstones and biliary duct stones are hypothesized to cause chronic inflammation leading to dysplasia. The exact mechanism whereby cholelithiasis causes/predisposes to gallbladder cancer remains debatable. Perhaps chronic mucosal damage due to mechanical forces exerted by the gallstone may be involved [8]. Between 0.5 to 1.5% of patients who undergo a simple cholecystectomy for presumed cholelithiasis are discovered incidentally to have gallbladder cancer [1]. Autopsy studies have revealed a 1-2% incidence of gallbladder carcinoma in patients with cholelithiasis [8].

#### 3.2.2. Chronic Inflammation

Chronic inflammation is considered a major factor in carcinogenesis, causing DNA damage, tissue proliferation, and cytokine and growth factor release. Another result of chronic inflammation is deposition of calcium within the gallbladder wall, causing the gallbladder to develop a bluish hue and become fragile—the “porcelain gallbladder.” While less than 1% of gallbladder specimens demonstrate this change, it is frequently (~25%) associated with gallbladder cancer. Only specimens with stippled calcification on imaging are considered potentially “premalignant” as transmural calcification is less likely to develop malignancy [7].

Chronic inflammatory diseases such as primary sclerosing cholangitis (PSC) are reported to be associated with a higher incidence of GBC. It is therefore recommended that patients with PSC should undergo annual gallbladder surveillance screening with ultrasound for the detection of any abnormal lesions [7].

Given the association between chronic cholecystitis and gallbladder cancer, it is questioned whether routine prophylactic cholecystectomy may be an effective way to prevent malignancy. Seretis et al. sought to answer this question by reviewing the prevalence of metaplasia in routine cholecystectomy specimens and found an increased prevalence of dysplastic changes, gallbladder wall thickening, and microlithiasis in specimens with metaplastic features. Microlithiasis is, however, more likely to be asymptomatic than macrolithiasis and therefore the practice of performing a cholecystectomy for all symptomatic cholecystitis patients may not be treating the population at the greatest risk as opposed to treating those with incidentally discovered microlithiasis on abdominal imaging [14].

#### 3.2.3. Gallbladder Polyps

While nearly 5% of all adults have gallbladder polyps, the majority are pseudopolyps with no neoplastic potential: cholesterolosis (60% gallbladder polyps), adenomyosis (25%), or inflammatory (10%) [7]. Other potential gallbladder polyps include nonneoplastic (hyperplastic and inflammatory) and neoplastic polyps (adenomas, leiomyomas, fibromas, and lipomas). Differentiating nonneoplastic from malignant/premalignant polyps is an important major preoperative diagnostic challenge [15]. Benign adenomas, constituting 4% of all gallbladder polyps, play an unclear role in neoplastic transformation; however, the absence of adenoma remnants in mucosa adjacent to adenocarcinoma suggests these tumours may not play a role in carcinogenesis in all cases [7].

Polyps at risk of malignant transformation are typically rapidly growing and >10 mm in size and solitary/sessile polyps in patients with gallstones of the age of 50+ years [7]. General consensus guidelines for removal of gallbladder polyps include polyps >10 mm in size, patients older than 60 years, increasing growth on serial imaging, and/or the presence of gallstones. These suggestions are, however, not firm evidence-based consensus guidelines. A recent study suggests that polyps larger than 2 cm are more likely to harbor high-grade dysplasia/malignancy and the authors concluded that all polyps >2 cm should be removed, whereas those <2 cm can be followed by serial ultrasound every 3–6 months [16]. By contrast, other authors point out that up to 40% of malignant gallbladder polyps may be <1 cm in size and thus patients with a polyp of 5–10 mm should not be excluded from investigation [17].

In summary, therefore, two treatment options are available for the treatment of polyps <10 mm: (i) cholecystectomy for symptomatic gallbladder polyps irrespective of size or (ii) serial ultrasounds until the polyp attains a size of ~10 mm [15].

#### 3.2.4. Pancreaticobiliary Maljunction Anomalies

Pancreaticobiliary maljunction (PBM) is an abnormal union of the biliary and pancreatic ducts located outside the duodenal wall in which a sphincter is not present. This congenital anatomic anomaly allows pancreatic fluids to reflux into the biliary system, causing chronic inflammation and genetic alterations, leading to increased cellular proliferation resulting in hyperplasia/dysplasia/carcinoma. This anomaly may be detected by cholangiography either with endoscopic retrograde cholangiopancreatography (ERCP) or magnetic resonance cholangiopancreatography (MRCP) or through endoscopic ultrasound (EUS) imaging [7]. EUS shows two thickened layers with epithelial hyperplasia and subserosal fibrosis, with or without a third layer containing a hypoechoic hypertrophic muscular layer [10]. Approximately 10% of patients with gallbladder cancer have this anomaly [7]. These patients also have a higher frequency of* Kras* mutations [18].

Among non-PBM patients, pancreaticobiliary reflux may occur secondary to a long common channel or high confluence of pancreaticobiliary ducts (HCPBD). A channel length greater than 8 mm is more frequent in patients with gallbladder cancer (38%) compared with normal gallbladders (3%) [10]. In these patients, it is thought that pancreaticobiliary reflux causes severe irritation of the gallbladder mucosa.

### 3.3. Exposures

A number of substances have been hypothesized to increase the risk of gallbladder cancer, including heavy metals and radon. It has been shown that patients with gallbladder cancer have significantly lower levels of selenium and zinc and higher levels of copper, lead, cadmium, chromium, and nickel in serum and bile compared to patients with cholelithiasis. Whereas selenium and zinc are antioxidants, the remaining heavy metals are well-recognized carcinogens [9, 19].

Workers in oil, paper, chemical, shoe, textile, and cellulose acetate fiber manufacturing have an increased risk of developing gallbladder cancer. Tobacco is also well recognized to be a significant risk factor. Drugs including methyldopa and isoniazid may additionally increase the risk of GBC. The risk associated with taking oral contraceptives remains controversial [7, 9, 19].

### 3.4. Infection

An association between* Helicobacter* infection of the bile and gallbladder carcinogenesis may be related to bacterial-induced degradation of bile acid; however, precise mechanisms remain poorly understood [6]. Liver flukes, particularly* Clonorchis sinensis* and* Opisthorchis viverrini*, have been implicated in cancer of the gallbladder [7].

Chronic bacterial cholangitis, usually due to* Salmonella* and* Helicobacter*, increases the risk of biliary tree malignancy. Colonization by bacteria may increase the risk of malignant transformation as the microorganisms degrade bile constituents by hydrolyzing bile salts and forming carcinogens. Chronic typhoid carrier status is thus a significant risk factor, with 6% of carriers developing this cancer (a 12x increased risk) [7].

## 4. Pathogenesis

Gallbladder cancer may arise in the gallbladder's fundus (60%), body (30%), or neck (10%) [20]. The development of gallbladder cancer is proposed to occur over a span of 5–15 years, with tissue alterations including metaplasia, dysplasia, carcinoma in situ, and invasive cancer [7].

The anatomy of the gallbladder is unique and predisposes the cancer to direct invasion as histologically the gallbladder wall is composed of a mucosa, lamina propria, smooth muscle layer, perimuscular connective tissue, and serosa: note the lack of submucosa in the gallbladder. Additionally, no serosa is present where the gallbladder attaches to the liver and, as such, direct infiltration of gallbladder cancer to the liver is the most common form of direct local spread [18, 21].

*Patterns of Spread*. Spread of gallbladder cancer occurs via four routes: (a) local invasion of the liver or other nearby structures, (b) lymphatic dissemination, (c) peritoneal spread, and (d) hematogenous spread. Direct extension of gallbladder cancer typically involves the liver (segments IV and V), bile duct, duodenum, colon, parietal wall, and/or abdominal viscera [1, 8]. Hepatic metastasis is most often the result of direct liver and portal tract invasion. Portal tract invasion can also be the result of lymphatic spread [22].

### 4.1. Molecular Pathogenesis

#### 4.1.1. Biological Pathways

Two distinct independent biological pathways based on morphological, genetic, and molecular evidence leading to gallbladder cancer are hypothesized: (1) a dysplasia-carcinoma sequence arising from metaplastic epithelium and (2) an adenoma-carcinoma sequence [23, 24]. 

*Theory #1*. In the chronically inflamed gallbladder, metaplasia is common, being present in over 50%. Similar to metaplasia of the stomach, gallbladder metaplasia occurs in two forms: gastric type and intestinal type [24]. Chronically inflamed gallbladders (both fluke-infested and sporadic) may express both pyloric gland and intestinal metaplasia; however, fluke-infested gallbladders more commonly express intestinal metaplasia and p53 mutations than sporadic gallbladder cancers [25]. However, the precise relationship between metaplasia and dysplasia remains ill-established.

The first theory suggests that dysplasia progresses to carcinoma in situ (CIS) which becomes invasive. This theory is supported by the finding that over 80% of invasive gallbladder cancers have adjacent regions of CIS and epithelial dysplasia [26]. One study demonstrated the presence of metaplasia, dysplasia, and CIS adjacent to the cancer in 66%, 81.3%, and 69%, respectively. Dysplastic lesions have molecular genetic evidence that supports progression towards CIS. It is well recognized that gallbladder dysplasia progresses to invasive cancer typically over a course of 15 to 19 years [27].

*Theory #2*. By contrast, less than 3% of early carcinomas have adenomatous remnants, suggesting this mechanism has limited importance in the carcinogenic pathway. There remains no way to predict which of these will undergo malignant transformation. Unlike well-established carcinogenic pathways in colorectal cancer [28], it remains debated in the literature whether or not adenomas are true precursors of invasive gallbladder carcinomas. Only 1% of cholecystectomy specimens have adenomatous polyps as preneoplastic lesions [6].

#### 4.1.2. Genetic Mutations

The precise genetic changes involved in the development of gallbladder cancer are poorly understood. A variety of genetic alterations are likely implicated in gallbladder cancer including oncogene activation, tumour suppressor gene inhibition, microsatellite instability, and methylation of gene promoter areas. Over 1281 genetic mutations have been identified in gallbladder cancer [23]. Specific genes implicated in carcinogenesis are summarized in Table 1 [23, 26, 29–37]. Early molecular changes are thought to include p53 mutation, cyclooxygenase-2 (COX2) overexpression, mitochondrial DNA mutations, and hypermethylation of promotors in tumour suppressor genes, with later events including inactivation of the fragile histidine triad (FHIT) and cyclin-dependent kinase inhibitor 2A (CDKN) tumour suppressor genes as well as loss of regions on chromosomes 9, 18, and 22. Dysplasia further leads to overexpression of p16 [23].

Like many malignancies,* Kras* and* TP53* are the best described genes implicated in gallbladder cancer. Carcinogenic pathways may include (1) inflammation secondary to gallstones leading to* p53* mutations and eventual carcinoma, (2) point mutation of* Kras* contributing to hyperplasia then carcinoma as seen in patients with an anomalous junction of pancreaticobiliary duct, and (3) neoplastic foci in gallbladder polyps secondary to* Kras *mutation [7].

Epigenetics may play a distinct role in gallbladder carcinogenesis. Methylation patterns of the tumour suppressor genes p16, APC, MGMT, hMLH1, RARbeta2, and p73 have been detected in 72% of GBCs and 28% of chronic cholecystitis, though rare in normal tissue [38, 39]. In keeping with the global prevalence variability, rates of methylation were compared in GBC patients from Chile versus the United States, and a significant difference in the methylation of APC (42% versus 13%) and p73 (14% versus 40%) was identified, suggesting a unique geographic-dependent biology. It is believed that the methylation level accumulates throughout the progression from chronic cholecystitis through the development of metaplasia [38].

The role of microsatellite instability (MSI) in the carcinogenesis of gallbladder cancer remains poorly described, with literature reporting MSI rates between 0 and 40% of cases; the largest and most recent study reported a prevalence of 7.8%. Moy et al. reported a strong correlation between global DNA methylation as measured by long interspersed element-1 (LINE-1) and loss of mismatch repair proteins suggesting that methylation causes silencing of these genes [40]. It is suggested that MSI may be more common in patients developing GBC secondary to abnormal anatomy and is not associated with Lynch syndrome. There is no reported significant difference in tumour grade, tumour stage, and overall survival in gallbladder cancer patients with or without MSI.

Loss of heterozygosity (LOH) has been described in a number of tumour suppressor genes in gallbladder cancer including chromosomes 1p34–36 (*p73*), 3p (*VHL*,* RAR-beta*,* RASSF1A*, and* FHIT*), 5q21 (*APC*), 8p21–23 (*PRLTS *and* FEZ1*), 9p21 (*p15*,* p16*), 9q (*DBCCR1*), 13q14 (*RB*), 16q24 (*WWOX *and* FRA16D*), and 17p13 (*p53*) [29].

Also associated with gallbladder cancers are sporadic reports that implicate lesser known genes. In gallbladder cancer, expression of ADAM-17 is increased in tumours with a high histological grade and pT stage as well as shorter overall survival [41, 42]. The ADAM gene family, of which the best known member is ADAM-17, has been implicated in regulation of ECM remodeling and cell migration. ADAM-17 (tumour necrosis factor-alpha converting enzyme, TACE) cleaves TNF-alpha from its precursor and releases EGFR ligands, amphiregulin, and heparin-binding epidermal growth factor (HB-EGF).

High mobility group protein A2 (HMGA2) is a nonhistone chromatin protein involved in tumorigenesis, invasion, and metastasis of tumours. Zou et al. showed statistically higher expression of HMGA2 in gallbladder cancer compared with normal tissue, polyps, and chronic cholecystitis. He also showed significantly decreased expression of CD9 in cancers compared to benign tissues [43]. Mobility related protein-1 (MRP1 aka CD9) is a glycoprotein that belongs to the transmembrane 4 superfamily and is related to tumour progression.

Thus, in summary, the pathogenesis of gallbladder cancer continues to be ill understood. As chronic inflammation is recognized as a key player in carcinogenesis causing DNA damage and tissue proliferation with cytokine and growth factor release, perhaps research may have to be undertaken in alternative pathways such as deciphering immune surveillance with special reference to intracellular and intercellular cell “chatter” which may be the earliest alteration that occurs in the carcinogenesis pathway. The field of cell signaling resulting in signal transduction in the immune system is yet to be explored in the pathogenesis of gallbladder cancers.

## 5. Staging

A number of staging systems have been described for gallbladder cancer including Nevin's staging system (Table 2) [44], the Japanese Biliary Surgical Society staging system (Table 3) [1, 45], and the TNM staging system of the American Joint Committee on Cancer (Table 4) [46].

## 6. Clinical Presentation

Gallbladder cancer typically presents in one of three ways: (a) malignancy suspected preoperatively, (b) malignancy discovered accidentally at cholecystectomy performed for presumed benign disease, and (c) malignancy diagnosed incidentally at pathological examination following routine cholecystectomy [6]. Over two-thirds of patients with gallbladder cancer are only diagnosed during surgery or postoperatively [47]. Symptomatic patients most commonly present with advanced disease, a truth that has not greatly changed in the past 85 years [48]. At presentation, gallbladder cancer is often similar to biliary colic or chronic cholecystitis. Right upper quadrant or epigastric pain is the most common symptom (54–83%), followed by jaundice (10–46%), nausea and vomiting (15–43%), anorexia (4–41%), and weight loss (10–39%) [1]. Jaundice may result either from direct invasion of the biliary tree or from metastatic disease to the hepatoduodenal ligament [6]. Only 3–8% of patients have a palpable mass [1]. Among patients who present symptomatically, tumours are typically advanced with 75% being nonresectable [6]. Among patients with a preoperative diagnosis of Mirizzi syndrome, 6–27.8% of patients will have a final diagnosis of gallbladder cancer [1].

Unsuspected gallbladder cancer is most commonly diagnosed incidentally after routine cholecystectomy. Lack of preoperative clinical suspicion and the absence of specific clinical or serological markers on history and physical exam are likely contributing factors for advanced stage diagnosis.

## 7. Diagnostic Imaging

### 7.1. Ultrasound

Ultrasonography is most frequently the initial diagnostic study obtained when gallbladder disease is suspected. On ultrasonography, gallbladder carcinoma may have one of three appearances: (1) a mass replacing or invading the gallbladder, (2) an intraluminal gallbladder growth/polyp, or (3) an asymmetric gallbladder wall thickening. In advanced disease, sensitivity and specificity of ultrasound imaging is 85% and 80%, respectively; however, in early disease, ultrasound examination often fails to detect any abnormality, particularly when the tumour is flat or sessile and is associated with cholelithiasis [6]. High-resolution contrast-enhanced ultrasonography accurately identifies up to 70–90% of polypoid gallbladder lesions [49]. Cholesterol pseudopolyps are typically pedunculated with a thin stalk, <1 cm in maximal diameter, and multiple in number with ultrasound findings of echogenicity without posterior acoustic shadowing. In contrast, malignant polyps are usually sessile, solitary, and >1 cm [20]. Contrast-enhanced ultrasonography with perflubutane has been described in which gallbladder cancer shows continuous staining throughout the tumour and an “eruption sign” [10]. Aside from its diagnostic utility, ultrasonography may provide information for disease staging by defining the extent of biliary tree involvement and confirming the presence of hepatic, arterial, or portal vein invasion.

*Endoscopic ultrasound (EUS)* is currently the definitive imaging modality in the staging of gallbladder cancer, allowing for precise imaging and acquisition of a fine needle aspiration (FNA) biopsy. Ultrasound- or CT-guided biopsy of mass lesions has a diagnostic accuracy of 80–90% [8]. A scoring system was devised by Choi et al. to predict the risk of neoplastic polyps, based on layer structure, echo patterns, polyp margin, polyp stalk, presence of gallstones, gender, age, and number of polyps, with a cut-off score of 6 conferring a sensitivity and specificity of 81% and 86% [50].

Newer technologies include contrast-enhanced harmonic EUS (CEH-EUS) to characterize gallbladder polyps. This modality has a greater accuracy than EUS, with a sensitivity of 93.5% and specificity of 93.2% [51]. They also include real-time elastography using acoustic radiation force impulse (ARFI) that uses high intensity focused ultrasound to determine tissue stiffness in a variety of organs, differentiating malignant from benign [10].

### 7.2. CT Scan

The most common evaluative imaging in gallbladder cancer is the CT scan, the utilization of which has been increasing over time [52]. CT scan may be useful in the diagnosis and staging of gallbladder cancer. This imaging modality may detect liver or porta hepatis invasion, lymphadenopathy, and involvement of the adjacent organs. Four patterns of gallbladder cancer have been described on CT scan: (a) a polypoid mass within the gallbladder lumen (15–25%), (b) focal wall thickening, (c) diffuse wall thickening (20% gallbladder cancers), and (d) a mass replacing the gallbladder (40–65%). These findings are, however, also features of inflammatory conditions such as xanthogranulomatous cholecystitis and adenomyomatosis, benign lesions, and metastatic disease [53]. Multidetector row CT (MDCT) may be used to further distinguish between malignant gallbladder wall thickening and benign gallbladder wall thickening, with 75.9% specificity and 82.5% sensitivity [10].

### 7.3. ERCP

Endoscopic retrograde cholangiopancreatography (ERCP) may demonstrate anomalous junction of pancreaticobiliary ducts and allows for the collection of bile samples, brush cytology, and/or intralesional biopsy [8]. ERCP is a poor tool for diagnosing gallbladder cancer as, while it accurately demonstrates filling defects, it does not delineate the surface of polypoid lesions. As such, it is best used for identifying tumour extension into the bile ducts.

### 7.4. MRI, MRA, and MRCP

The combination of MRI (magnetic resonance imaging) with MRA (magnetic resonance angiography) and MRCP (magnetic resonance cholangiopancreatography) is useful in detecting vascular invasion (100% sensitivity and 87% specificity), biliary tract involvement (100% sensitivity and 89% specificity), liver invasion (67% sensitivity and 89% specificity), and lymph node involvement (56% sensitivity and 89% specificity) [6]. MRI has been shown to be superior to CT scan for differentiating T1a lesions from T1b or greater and as such may be useful in preoperative management planning [54].

The early and prolonged enhancement of malignant lesions differs from the early enhancement with subsequent washout of benign masses. Similar patterns of enhancement can assist in differentiating malignant wall thickening from benign wall thickening along with an irregular versus a smoothly delineated enhancement, respectively [55]. Differentiation of gallbladder cancer from adenomyomatosis may be challenging as the latter can also present with focal or diffuse wall thickening. Intramural cyst-like spaces due to dilated Rokitansky-Aschoff sinuses on MRI, the “pearl-necklace appearance,” are indicative of adenomyomatosis, though they are not necessary to make this diagnosis [20, 56].

Addition of* diffusion-weighted imaging (DWI)* may aid in the differentiation of malignant from benign gallbladder disease as it provides a greater sensitivity [56]. Addition of DWI to standard T2WI improves the sensitivity, specificity, positive predictive value (PPV), and negative predictive value (NPV) from 97.2%, 86.7%, 74.5%, and 98.7% to 97.2%, 92.2%, 83.3%, and 98.8% [55].

### 7.5. FDG-PET Scan

PET scanning may be useful in diagnosing ambiguous primary lesions, detecting residual disease after cholecystectomy, and uncovering distant disease not otherwise appreciable by other imaging modalities. Given the high incidence of metastatic disease, PET scan is a useful preoperative imaging modality. Indeed nearly 25% of patients with gallbladder cancer who underwent preoperative PET scanning had a change in their operative management in one study [52].

FDG-PET scanning with CT (PET/CT) combines metabolic and anatomical localizations of suspicious lesions. It has been shown to detect 95.9% of primary gallbladder cancers, 85.7% of lymph node involvement, and 95.9% of metastatic disease [4]. This combination of diagnostic imaging can be used (a) preoperatively to define the possibility of curative surgery and (b) in patients postoperatively for restaging. The authors reported a negative predictive value of 100%, indicating a negative study that excludes the presence of malignancy; false positives were due to inflammation secondary to cholecystitis, RAS, or adenomyomatosis [4]. Elevated CRP may negatively affect the accuracy of this imaging modality [57].

### 7.6. Percutaneous Approaches

Percutaneous transhepatic fine needle aspiration and percutaneous transhepatic cholecystoscopy may be used in the evaluation of gallbladder polyps. While these modalities portend an accurate diagnosis, they are time consuming, more invasive, and poorly tolerated by the patient [49]. Image-guided FNA including ultrasound-guided or CT-guided biopsy has the potential for a diagnostic accuracy of 80–90% [8]. False-negative results of 11–41% may be attributable to incorrect sampling, necrosis, or fibrosis. FNA can be used to detect the uncommon variants of gallbladder cancer [58].

## 8. Pathology

### 8.1. Gross Pathology

Gallbladder cancer may present as a mass lesion, localized wall thickening with induration of the wall, or polypoidal growth. Obstruction of the neck and/or cystic duct may cause distension or collapse of the gallbladder; neoplasms in the body may constrict the lateral wall resulting in an hour-glass deformity. These lesions are typically grey-white in colour; however, mucinous and signet ring lesions have a gelatinous cut surface.

### 8.2. Cytopathology

A recent study by Yadav et al. described the cytopathology of various subtypes of gallbladder cancer as follows:

- Papillary adenocarcinoma: papillae with vascular core and minimal pleomorphism.
- Mucinous adenocarcinoma: single cells or clusters with >50% extracellular mucin.
- Signet ring cell carcinoma: a predominance of signet ring cells.
- Adenosqumous carcinoma: an admixture of glandular and squamous components.
- Squamous cell carcinoma: atypical keratinized cells and/or polliwog cells in a necrotic background.
- Neuroendocrine carcinoma: rosettes, salt/pepper chromatin, anisonucleosis, and/or nuclear molding
- Small cell carcinoma: smudge cells, scant cytoplasm, necrosis, salt/pepper chromatin, and/or nuclear molding
- Undifferentiated carcinoma NOS: dispersed and highly pleomorphic cells with abundant necrosis [58].

### 8.3. Histopathology

Adenocarcinoma is the most common histologic type, accounting for 98% of all gallbladder tumours, two-thirds of which are moderately/poorly differentiated. The remaining common histopathological variants include papillary, mucinous, squamous, and adenosquamous subtypes [7]. Other rare types of gallbladder cancer include carcinosarcoma, small cell carcinoma, lymphoma, signet ring cell-type tumours, and metastases [59]. Tumours may contain more than one histological variant [60].

Though most traditional gastrointestinal adenocarcinomas are classified as either differentiated or undifferentiated, biliary tract cancer is predominately a well-differentiated adenocarcinoma with a minor component of poor differentiation, thus allowing venous, lymphatic, and perineural invasion in “advanced disease” that is not as readily apparent in “early” biliary cancers. Intramural invasion can be classified as either infiltrative growth-type or destructive growth-type, the latter conferring a worse overall prognosis [61].

As extensive tumour necrosis with minimal residual viable tumour can mimic acute gangrenous cholecystitis, adequate sampling is critical. Features of cholecystitis such as edema, vascular congestion, hemorrhage, and fibrin deposition to the adventitia/muscle may help in identifying a benign process [60].

Distinguishing between well-differentiated adenocarcinoma and Rokitansky-Aschoff sinuses (RAS) can be challenging as RAS can extend deep into the perimuscular adipose tissue and be located throughout the gallbladder. Desmoplasia does not rule out RAS as it may surround RAS particularly in the setting of chronic cholecystitis. Adenomyosis can mimic gallbladder adenocarcinoma as it is characterized by epithelial proliferation with deep diverticulae extending into the muscular layer; however, the glands are cytologically bland with cystic dilatations that communicate with the lumen of the gallbladder [60].

Immunohistochemistry of gallbladder adenocarcinoma is similar to that of bile duct and pancreatic carcinoma. These tumours are positive for cytokeratin 7 (CK7) with focal expression of carcinoembryonic monoclonal antibody (CEA-M), CA19-9, MUC1, B72.3, and MUC5AC [60].

The remaining 2% of gallbladder cancers include the following:

- *Papillary Adenocarcinoma*. It represents ~5% of gallbladder cancers. Two subtypes of papillary adenocarcinoma have been described: invasive and noninvasive [62]. On histologic examination, these tumours typically consist of fibrovascular stalks lined by malignant epithelial cells, often with the production of mucin in the gallbladder. Noninvasive papillary tumours have a tendency towards intraluminal growth, filling the gallbladder prior to locoregional invasion, and are therefore typically associated with a better prognosis than routine gallbladder cancers [59]. Regardless of size and degree of differentiation, these do not metastasize and are best treated with a simple cholecystectomy. In contrast, invasive papillary adenocarcinoma is associated with a 10-year relative survival rate for tumours confined to the gallbladder wall of 52% and <10% among those with lymph node metastases [62].
- *Mucinous Adenocarcinoma*. Defined as a carcinoma with >50% stromal mucin deposition, it comprises 2.5% of all gallbladder cancers and is very poorly described in the literature. These tumours have less preponderance for females (1.1) and typically present with an initial diagnosis of acute cholecystitis. They are typically larger than adenocarcinoma (4.8 cm versus 2.9 cm). These tumours are usually mixed mucinous rather than pure colloid [63]. The presence of abundant mucin on radiographs (i.e., spotty and hyperechoic contents on sonography) is diagnostic [59]. Differentiation between mucinous carcinoma of the gallbladder and pseudomyxoma peritonei is challenging [60]. The tendency of mucinous adenocarcinoma towards invasive growth confers a poor prognosis.
- *Signet Ring Cell Carcinoma*. It is identified by intracytoplasmic mucin displacing the nuclei to the periphery. These tumours characteristically have infiltrative submucosal growth patterns resembling* linitis plastica* of the stomach. On sonography and CT, these tumours show an echogenic polypoid mass with target-like wall thickening [59].
- *Squamous/Adenosquamous Cell Carcinoma (SC/ASC)*. Its incidence ranges from 1.4–12.7% [59]. There remains no consistent definition in the literature outlining the extent of squamous differentiation required to categorize a tumour as “adenosquamous” carcinoma rather than adenocarcinoma [64]. These tumours typically arise from the gallbladder fossa and present with rapid and aggressive growth. Adenosquamous carcinoma may show comedo-like necrosis with associated tumour giant cells. Pure squamous cell carcinoma often shows prominent keratinization. Due to the rarity of this lesion, exact treatment protocols and outcome data are controversial in the published literature. Residual disease is established as a significant independent prognostic factor for these tumours [65].
- *Cribriform Carcinoma*. It accounts for <1% of all gallbladder carcinomas and is thought to occur in younger patients. The histopathological characteristics are highly reminiscent of mammary gland cribriform carcinoma and as such this diagnosis must be excluded. The presence of bonafide “comedonecrosis” may help accurately distinguish primary gallbladder cribriform carcinoma from metastatic breast cancer. Lack of estrogen and progesterone receptor immunoreactivity may aid in differentiating primary gallbladder cribriform carcinoma from its counterpart metastatic breast lesion [66].
- *Hepatoid Adenocarcinoma*. It is characterized by foci of both adenomatous differentiation and hepatocellular differentiation of the gallbladder with a natural history similar to hepatocellular carcinoma. On histopathology, these tumours are composed of large or polygonal cells with an abundant eosinophilic cytoplasm with or without medullary proliferation. On immunohistochemistry, hepatoid adenocarcinomas may express alpha-fetoprotein (AFP), albumin, transferrin, PIVKA, and alpha-1-antitrypsin. While AFP remains the most important marker of this lesion, not all hepatoid adenocarcinomas are positive for AFP. These tumours must be differentiated from hepatocellular carcinoma invasion into the gallbladder [67].
- *Clear Cell Adenocarcinoma*. It is exceedingly rare and is often identified with other components such as adenocarcinoma, adenosquamous carcinoma, or mucinous carcinoma. On histopathology, clear cell adenocarcinoma (CCA) has an infiltrative growth pattern with or without glandular differentiation, composed of polygonal/cuboidal clear cells with minimal cytological atypia [68]. CCA of the gallbladder should be differentiated from a metastases most commonly from the kidneys [68, 69].
- *Undifferentiated Carcinoma*. It can present as four histologic variants: (i) spindle and giant cell type, (ii) osteoclast-like giant cell type, (iii) small cell type, and (iv) nodular or lobular type. These tumours characteristically lack glandular structures [70]. Spindle cell carcinoma (SpCC) of the gallbladder is composed predominately of sarcomatous elements with areas of carcinomatous differentiation and demonstration of this biphasic appearance is essential for diagnosis. On immunohistochemistry, SpCC will usually demonstrate biphasic reactivity to cytokeratins (CK, EMA) and mesenchymal antibodies such as vimentin. This tumour confers a worse prognosis compared with gallbladder adenocarcinoma [71]. Giant cell type carcinomas are assumed to arise when there is dedifferentiation of a preexisting well-differentiated adenocarcinoma to anaplastic giant cell components [70].
- *Gallbladder Sarcoma*. It is exceedingly rare and patients present similarly to gallbladder adenocarcinoma. Tumour types include leiomyosarcoma, rhabdomyosarcoma, angiosarcoma, Kaposi's sarcoma, malignant fibrous histiocytoma, synovial sarcoma, malignant GIST, and liposarcoma. Though the pathogenesis of these tumours remains unclear, gallbladder sarcomas are hypothesized to arise from totipotential stem cells or paramesonephric tissue [72]. Gallbladder carcinosarcoma is rare and very aggressive as it spreads by direct invasion, hematogenously, and via the lymph nodes [73]. The mean survival after diagnosis is measured in months.
- *Neuroendocrine Tumours*. Neuroendocrine Tumours of the gallbladder comprise only 0.5% of all neuroendocrine tumours and ~2% of gallbladder cancers. These tumours are thought to derive from multipotent stem cells, as normal gallbladder mucosa does not contain neuroendocrine cells, though mucosa undergoing gastric/intestinal metaplasia can express a variety of neuroendocrine hormones including serotonin, histamine, gastrin, somatostatin, and glucagon. Virtually all neuroendocrine tumours of the gallbladder reported have coexisting gallstones with chronic cholecystitis with less than 1% of patients presenting as functioning lesions such as carcinoid syndrome [74] and/or hyperglycemia [75]. Some authors suggest these lesions should be treated similarly to gallbladder adenocarcinoma, while others recommend a more aggressive approach. They are typically identified at an advanced stage, with a 5-year reported survival rate of ~20% [74]. Neuroendocrine tumours are classified according to their differentiation as carcinoid tumours (well differentiated) or small cell carcinoma (poorly differentiated).

*Carcinoid tumours* are rare with differentiation between them and carcinoma preoperatively being often impossible as imaging features are similar. Patients typically present with vague symptoms, and only 3.3–3.7% present with carcinoid syndrome [76, 77]. On histopathological examination, atypical variants may have cellular atypia and mitosis which are associated with a worse prognosis [77]. Immunohistochemistry is useful in the accurate identification of carcinoids, with positivity for neuroendocrine markers [76]. A SEER database review reported a 10-year survival of 36% [78].

*Small cell carcinoma (SCC)* of the gallbladder is extremely rare, comprising only 0.5% of all gallbladder cancers [79]. These patients may present with paraneoplastic syndromes including Cushing's syndrome and sensory neuropathy [80]. These tumours are most common in elderly females, particularly those with cholelithiasis [79]. Gallbladder SCC usually presents as a large mass containing extensive necrosis with a marked propensity for invasive submucosal growth. On histopathology, ~72% are pure SCC and the remaining 28% are mixed SCC + adenocarcinoma or squamous cell carcinoma [81]. Unlike adenocarcinoma, SCCs are bulky tumours with local invasion, paraneoplastic leukocytosis, and/or hypercalcemia and are associated with metastases and worse prognosis [82]. Based on the SEER database, gallbladder SCC has essentially no survivors at 10 years [78].

## 9. Surgical Treatment

Complete surgical tumour resection is the only curative treatment for GBC. A complete resection is often challenging as the gallbladder has anatomically neighboring vital structures such as the porta hepatica, and this malignancy has a propensity for hepatic invasion with early lymphatic metastases. The “radical cholecystectomy” was first proposed by Glenn and Hays in 1954 in which the gallbladder bed with a rim of liver tissue and lymphatic tissue within the hepatoduodenal ligament were excised en bloc [83]. An “extended radical cholecystectomy” that was proposed in 1982 differs in that the lymphatic tissue within the hepatoduodenal ligament, the posterosuperior head of the pancreas, with dissection around the portal vein, and common hepatic artery are removed en bloc with the gallbladder, a rim of liver tissue, and the extrahepatic bile duct [83]. During surgical resection, it is imperative to avoid incising the gallbladder or spilling its contents as this is associated with increased morbidity and mortality.

Prior to definitive management by laparotomy, staging laparoscopy is often helpful to assess for peritoneal spread or discontiguous liver disease. Weber et al. reported that unresectable disease was identified in 48% of their study patients by laparoscopy, thereby preventing unnecessary morbidity with open laparotomy [6]. While many recommend routine diagnostic laparoscopy for all gallbladder cancer-directed operations, there are authors that suggest staging laparoscopy is a waste of healthcare resources. However, when a gallbladder cancer is suspected preoperatively, laparoscopic cholecystectomy is contraindicated.

The extent of surgical intervention may range from simple cholecystectomy to being combined with partial hepatectomy, with or without regional lymph node dissection. At a minimum, definitive surgery includes removal of involved liver parenchyma as well as regional lymph nodes. While the appropriate surgical intervention may be estimated using TNM staging (Table 4) [84], there remains a paucity of randomized data to definitely guide management.

### 9.1. Incidental/Unsuspected Gallbladder Carcinoma

Incidental identification of gallbladder cancer occurs in 0.2–3% of all cholecystectomies for presumed benign disease [85]. In fact only 30% of patients with gallbladder cancer are suspected of harbouring a malignancy preoperatively [86]. Intraoperative findings that may indicate gallbladder cancer include ulcerations or small plaques on gross examination of the opened specimen or a firm mass in more advanced tumours. It has been proposed that ideally all cholecystectomy specimens should be opened and examined intraoperatively. If gallbladder cancer is suspected at routine laparoscopic cholecystectomy an intraoperative frozen section should be sent to the pathology laboratory for immediate tissue confirmation. Features indicating the need for intraoperative frozen section include (a) macroscopically contracted or sclerotic mucosa, (b) thickening of one part of the gallbladder wall, (c) normal tissue replaced by connective tissue in all wall layers, (d) macroscopic mucosal color change, and/or (e) presence of a polypoid lesion. Sensitivity and specificity of intraoperative frozen section are reported at 90% and 100%, respectively [84]. Though frozen section may be unable to differentiate carcinoma in situ from epithelial atypia, it has a 70–86% accuracy rate in determining the depth of invasion of the carcinoma [86].

The decision whether or not to convert to open laparotomy with a positive frozen section remains controversial, and no consensus guidelines have been established. Indications for conversion to open laparotomy include difficult dissection or a high risk of gallbladder rupture. Some surgeons advocate for immediate conversion to radical resection including port sites. As T1a lesions are treated with a simple laparoscopic cholecystectomy, and T3/T4 lesions should have been preoperatively diagnosed with imaging, it is the T1b and T2 lesions that are most commonly encountered unexpectedly intraoperatively and cause this management dilemma. An incomplete oncologic operation is undesirable, yet so is major resection for lesions that are ultimately benign or diffusely metastatic [84]. It is recently suggested that T1b tumours are best treated with a wedge resection of 2-3 cm of the gallbladder bed with lymph node dissection of the hepatoduodenal ligament with the initial cholecystectomy. Radical reresection of these tumours confers a survival benefit of 60–100% [86]. Other studies have, however, found no improved prognosis in patients with pT1b tumours treated with radical resection [87]. Tumours that invade the subserosal layer (T2+) require reoperating for radical surgery to improve survival. These patients require resection of liver segments IVb and V [86]. Evaluation of the initial cystic duct margin guides surgical management: a negative margin spares the biliary tree and a lymphadenectomy with IVb/V liver resection is performed. By contrast, a positive margin requires intraoperative identification of the cystic duct with resampling and resection of the common duct with portal nodes and liver bed [47].

After cholecystectomy, time to recurrence is generally rapid, with a mean of 4 months [8]. The ideal timeframe between cholecystectomy and radical surgery for incidentally discovered gallbladder cancer remains ill-defined; however, some authors suggest delayed referral to a tertiary center after cholecystectomy is not a risk factor for finding inoperable disease [88]. It is however suggested that reresection should be performed within 10 days of the initial surgery [86].

### 9.2. Port Site Recurrences

Port site recurrence is traditionally a major concern, reported in 14–29% of patients within 6–10 months. This risk is elevated in the event of gallbladder perforation at a rate of 40%. The precise mechanism responsible for port site recurrence remains ill understood. Theories include (a) direct mechanical contamination—tumour cells left at the site during tissue retrieval or removal of contaminated instruments, (b) indirect mechanical contamination due to leakage of gas along the trocars (chimney effect), (c) changes in the host immune response, (d) hematogenous dissemination, and (e) surgical technique [1]. Use of a retrieval bag in all laparoscopic cholecystectomies is recommended to prevent recurrences as it is not always possible to foresee problems with retraction and, should the gallbladder rupture, it is preferable to do so in a retrieval bag [47].

One-year survival rate among patients with port site recurrence is <30% [87]. Though port site seeding is associated with peritoneal carcinomatosis and is a poor prognostic factor, port site excision does not improve survival. A recent study by Fuks et al. retrospectively reviewed 218 incidentally discovered gallbladder cancers after laparoscopic cholecystectomy and concluded that port site excision did not improve survival and as such should not be routinely advocated during definitive surgical treatment. Poor prognosis in these patients may be due to several factors that include the following: these tumours are advanced stage, lymph node involvement is nearly exclusive (100% and 92% in two series), and concomitant peritoneal carcinomatosis is common [85].

### 9.3. Tis/T1a Disease

Tis/T1 gallbladder cancer is typically diagnosed after cholecystectomy. In those with Tis and T1a, simple cholecystectomy is sufficient therapy [84]. Specific attention to the cystic duct margin is, however, imperative as this remains the most important prognostic factor in these early cancers. Intraepithelial extension into Rokitansky-Aschoff sinuses (RAS) significantly shortens the survival and is an independent prognostic factor. Some authors suggest that RAS involvement may indicate the need for additional radical surgery [89]. In the absence of gallbladder perforation, port site excision is not indicated [84]. With a simple cholecystectomy, the 5-year survival rate is reported at 100% with no possible benefit of more aggressive surgical management; however, unfortunately few cases of gallbladder cancer are identified at this early stage [2].

### 9.4. T1b Disease

Management of T1b tumours that invade the muscular layer of the gallbladder remains controversial. While some authors maintain simple cholecystectomy is adequate in this population with 5-year survival up to 100%, locoregional recurrence has been well reported with 5-year survival rates as low as 37.5–68% [1]. Up to 30–60% of patients treated with simple cholecystectomy will have recurrence [84]. A 21-year analysis of stage I GBC recommends review of extensive resection (cholecystectomy + lymph node dissection or radical cholecystectomy) as it allegedly improved disease-specific survival when compared with a simple cholecystectomy [90]. As such, some authors recommend extended cholecystectomy including wedge resection of the gallbladder bed with segment IVb and V resection and/or N1 lymph node dissection. Other authors have failed to show any survival improvement with the addition of liver wedge resection/common bile duct resection/pancreaticoduodenectomy compared with a cholecystectomy and hepatoduodenal lymph node dissection [91]. Lymphatic metastases are more common than in T1a, with 20% of patients having nodal and 28% lymphovascular disease [84].

### 9.5. T2 Disease

Simple cholecystectomy is insufficient in the treatment of T2 disease as it confers a 5-year survival rate of only 20–40% [6]. One large study found a nearly threefold increase in median survival among patients with T2 disease who underwent radical resection compared with simple cholecystectomy [52]. Radical cholecystectomy with wedge resection of the gallbladder bed (or segments IVb and V) and regional lymph node dissection are therefore necessary in the treatment of T2 disease. The use of en bloc resection increases five-year survival to over 80–90% [2, 6]. The extent of hepatic resection depends on involvement of the major hepatic arterial or portal venous structures. Involvement of the right portal pedicle necessitates a right hepatectomy; however, in its absence, resection of segments IVb and V is adequate [6]. In many centers, bile duct resection and reconstruction are standard for T2 gallbladder cancers; however, there remains a paucity of literature to support this practice [6]. In T2 disease, the rate of lymph node metastases is 19–62% [1]. The optimal extent of lymph node resection remains undetermined.

### 9.6. T3/T4 Disease

The best management of advanced gallbladder cancer remains a challenge for tumours that invade the serosa and/or adjacent organs (T3) and those that invade the main portal vein or hepatic artery or two or more extrahepatic organs/structures (T4). The morbidity and mortality of aggressive surgical management compared with the potential survival benefits remain unclear. Factors that may preclude extensive surgery in patients with advanced disease include poor physiologic status, the extent of the disease, and the presence of comorbidities. As such, surgical resection is only recommended when there is potential for a curative R0 resection.

In T3 tumours with direct invasion to adjacent duodenum, stomach, or colon, surgical resection is indicated. These tumours are usually amenable to a radical resection; however, such intervention is associated with a high degree of morbidity. Five-year survival rates for T3 gallbladder cancer range from 30–50% [6].

Management of hepatic invasion is inconclusive. No significant difference in survival was found between patients treated with gallbladder bed resection and those who underwent a formal segmental IVa + V hepatectomy [92].

The National Comprehensive Cancer Network (NCCN) recommends that tumours with T1b, T2, and T3 tumours should undergo radical reoperation including hepatic resection and lymph node dissection with or without common bile duct resection and reconstructive hepaticojejunostomy. Poor compliance, however, with these guidelines has been described, with only 13% and 6.9% of patients receiving radical repeat resections/hepatectomy and lymphadenectomy, respectively [52].

T4 tumours are typically unresectable, and palliation is indicated. Criteria for nonresectability include metastatic disease, involvement of main portal vein or hepatic artery, involvement of the portal vein or hepatic artery branches of both lobes of liver, simultaneous involvement of ipsilateral hepatic artery and contralateral portal vein, simultaneous involvement in both lobes of liver at the level of confluence of segmental bile ducts to form hepatic ducts, and contiguous involvement of more than 2 segments each in both lobes of liver. These criteria are, however, not binding. For example, two exceptions in the literature are the following: (a) if main portal vein invasion is present, then portal vein resection and reconstruction may be indicated rarely or (b) if there is extensive extrahepatic organ involvement this may be resected en bloc [6]. Traditionally, paraaortic lymph node metastasis was a contraindication to surgical resection; however, it is reported that resection in these patients confers a similar survival to those with isolated liver metastases and better survival than unresected tumours with other sites of metastatic spread. Contraindications to surgical resection in advanced tumours however remain poorly defined and as such each case should be evaluated on an individual basis [93].

The combination of hepatectomy and pancreaticoduodenectomy is commonly indicated when there is direct duodenal or pancreatic invasion and peripancreatic lymph node involvement. The presence of peripancreatic nodal involvement is not a contraindication for pancreaticoduodenectomy for gallbladder cancers provided an R0 resection is feasible. This procedure provides the greatest number of dissected lymph nodes [83]. The survival benefits of these procedures remain ill defined and geographically dependent. As such, aggressive surgery is not currently recommended routinely in this particular scenario.

### 9.7. Liver and Bile Duct Resection

Typically hepatic invasion occurs initially to segments IV and V. The extent of hepatectomy is dictated by the T-stage, the anatomical location, and size of the tumour. A surgical tumour-free margin of 2 cm is required; however, the extent of liver resection for T1b or higher tumours remains controversial. Some authors maintain that hepatic wedge excision is not appropriate for T1b/T2 disease as significant bleeds and bile leaks may result from an inconsistent thickness of tissue around the gallbladder. If there is invasion of the liver hilum, right hepatectomy with or without bile duct resection or portal vein resection is necessary for a curative intent. The operative mortality rate for extended radical surgery is <5% [1].

In the instance of a prior cholecystectomy with indications for a second operation, the status of the cystic duct margin is of utmost importance. If negative for malignant cells, the biliary tree may be preserved and a lymphadenectomy with IVB and V gallbladder bed liver resection is indicated. In contrast, a positive margin necessitates intraoperative identification of the cystic duct with resampling. If this sampling is positive, or the cystic duct stump cannot be identified, resection of the common duct, portal nodes, and liver bed to optimize surgically negative margins with Roux-en-Y hepaticojejunostomy is recommended. While some surgeons advocate for elective bile duct resection to improve node clearance, no survival benefit has been identified with this additional surgery [2].

### 9.8. Lymph Node Dissection

Regional lymph nodes of gallbladder cancer are classified based on the nodes involved: N1 (cystic, pericholedochal, and hilar lymph nodes, hepatoduodenal ligament), and N2 (peripancreatic (head only), peridutal, periportal, common hepatic artery, coeliac, and superior mesenteric artery lymph node). For T2–T4 disease, N1 and N2 regional lymph node dissection is indicated [1]. The most commonly involved initial nodes are the cystic and pericholedochal. It is strongly recommended that formal portal lymphadenectomy includes the nodal tissue of the hepatoduodenal ligament and portacaval and retroduodenal regions [2]. There remain no randomized control trials comparing survival with the extent of lymph node dissection [1]. It has been shown, however, that radical lymph node dissection is effective in up to three positive lymph nodes provided a R0 resection is attainable [83].

As the most powerful predicting factor for survival is nodal status, effective lymph node dissection is reported to be the most valuable procedure for improving survival. Adequate assessment of lymph node involvement, per the American Joint Committee on Cancer (AJCC) guidelines, recommends resection and pathologic examination of a minimum of three regional lymph nodes (cystic, pericholedochal, retroportal, periduodenal, peripancreatic, coeliac, and superior mesenteric nodes) [90]. A survival advantage is reported when three or more nodes are removed compared with 2 or less, with overall median survival in one study of 18 versus 5 months, respectively [52]. Significant differences in survival are reported between node-negative and node-positive disease, with 5-year survivals of 58–77% versus 0–45%, respectively. Regional lymph node involvement in T2 and T3/T4 tumours occurs in 19–62% and 75–85% and N2 lymph node involvement occurs in 18–36% and 42–71% [1].

## 10. Medical Treatment

**Figure 1:**
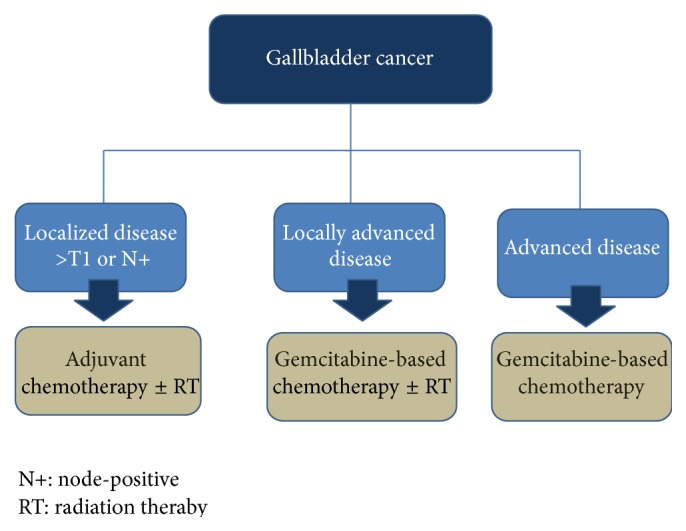
Role of systemic therapy in the management of gallbladder cancer.

Systemic therapy is used in curative and palliative setting in the management of gallbladder cancer in 3 situations: (1) in adjuvant therapy alone or in combination with radiation following surgical resection, (2) in locally advanced nonmetastatic unresectable disease alone or in combination with radiation therapy, and (3) in advanced metastatic disease (Figure 1).

There is a paucity of randomized controlled studies in the management of gallbladder cancer in relationship with systemic therapy due to the rarity of gallbladder and other biliary tract cancers. Most studies are inclusive of all biliary tract cancers and there are very few gallbladder cancer-specific studies.

### 10.1. Resectable Gallbladder Cancer

#### 10.1.1. Adjuvant Therapy

Evidence regarding adjuvant therapy in gallbladder cancer with few exceptions is mostly limited to retrospective studies. Most studies were comprised of small, heterogeneous groups of patients seen at a single institution. Several retrospective series and small phase II studies suggest better survival in patients who receive postoperative adjuvant treatment. The only phase III randomized trial regarding benefit of adjuvant therapy in gallbladder and biliary tract cancer is reported by the Japanese group. In this phase III multicenter randomized trial, 508 patients with resected pancreaticobiliary cancer were randomly assigned two cycles of intravenous mitomycin and 5-FU (MF) followed by maintenance oral 5-FU until disease recurrence versus observation. In a subgroup of 140 patients with gallbladder cancer, 5-year disease-free survival (DFS) rates of patients treated with adjuvant MF was 20.3% compared with 11.6% with observation (*P* = 0.02). The 5-year survival rate was significantly better in the adjuvant therapy group (26.0%) compared with the control group (14.4%) (*P* = 0.03) [94].

A meta-analysis which included 20 studies involving 6712 patients assessed the impact of chemotherapy, radiation therapy, or both therapies as an adjuvant to curative-intent surgery for the management of biliary tract cancers comprising extrahepatic and gallbladder cancers. Of 6712 patients 4915 were treated with surgery alone, and 1797 received adjuvant therapy. The meta-analysis reported a nonsignificant improvement in overall survival with any adjuvant therapy compared with surgery alone (odds ratio (OR), 0.74; *P* = 0.06). The association was significant when the two registry analyses were excluded. A nonsignificant benefit was also observed when disease sites were analyzed independently (gallbladder: OR, 0.81; 95% CI, 0.49 to 1.35; *P* = 0.41). The benefit of adjuvant therapy was dependent on treatment modality. Patients who received chemotherapy (OR, 0.39; 95% CI, 0.23 to 0.66; *P* < 0.001) or chemoradiotherapy (OR, 0.61; 95% CI, 0.38 to 0.99; *P* = 0.049) derived greater benefit than patients who were treated with radiation therapy alone (OR, 0.98; 95% CI, 0.67 to 1.43; *P* = 0.90). Nine studies reported nodal or margin positivity. Pooled data revealed a significant benefit for adjuvant chemotherapy or chemoradiation treatment (*n* = 230) in node-positive disease (OR, 0.49; 95% CI, 0.30–0.80; *P* = 0.004) or in cancers with R1 disease (OR, 0.36; 95% CI, 0.19 to 0.68; *P* = 0.002) [95]. An exploratory analysis that demonstrated greater magnitude of benefit from adjuvant therapy in studies included patients with node-positive disease, R1 disease, or both diseases compared to studies that did not include patients with node-positive or R1 disease. Similar findings were also seen in Surveillance, Epidemiology, and End Results- (SEER-) based study that was not included in this meta-analysis. This study demonstrated that, with the exception of T1N0 patients, 6 months of chemotherapy or radiation after surgery was associated with a better survival [96].

Even though the meta-analysis favors adjuvant therapy in patients with high risk, that is, node-positive gallbladder cancer, it does not resolve the question of the benefit of adjuvant therapy in patients with low risk disease. Moreover, the best treatment strategy, for instance, chemoradiotherapy versus chemotherapy alone, in adjuvant setting is not known.

The National Comprehensive Cancer Network (NCCN) guidelines for gallbladder cancer support adjuvant fluoropyrimidine chemoradiation or fluoropyrimidine or gemcitabine chemotherapy in patients with >T1N0 gallbladder cancer following curative surgery (National Comprehensive Cancer Network). Furthermore, the European Society of Medical Oncology (ESMO) guidelines also suggest consideration of postoperative chemoradiotherapy in patients with high risk gallbladder cancer [97].

The results of several phase III, randomized controlled trials evaluating the benefit of adjuvant chemotherapy are awaited. These trials included patients with completely resected biliary tract and gallbladder cancers. The United Kingdom trial [98] randomly assigned patients to eight cycles of capecitabine versus observation. The accrual for this study is completed. The French trial [99] that randomly assigns patients to 12 biweekly cycles of gemcitabine plus oxaliplatin versus observation is currently recruiting patients. ACTICCA-1 is a multicentre German phase III trial which is evaluating 24 weeks of gemcitabine and cisplatin after curative resection of biliary tract and muscle invasive gallbladder cancers (adjuvant chemotherapy with gemcitabine and cisplatin compared to observation after curative intent resection of biliary tract cancer [100]).

Given the poor prognosis of patients with gallbladder cancer with T ≥ 2 and/or node-positive disease, we recommend adjuvant therapy for such patients. As higher stage gallbladder cancers have a high incidence of both local failure and distant failure after surgical resection, despite limited evidence, a locoregional adjuvant treatment can be considered similar to other extrahepatic biliary cancers. The optimal adjuvant therapy is unknown though six months of gemcitabine or fluoropyrimidine-based chemotherapy with or without fluorouracil-based chemoradiation can be considered.

#### 10.1.2. Neoadjuvant Therapy

Currently outside the setting of a clinical trial neoadjuvant therapy is not recommended for surgically resectable gallbladder cancer. Trials with a neoadjuvant strategy may provide opportunities for the development of predictive markers to guide personalized treatment in patients with gallbladder and biliary tract cancer.

#### 10.1.3. Follow-Up after Curative Therapy

There is lack of level 1 evidence with respect to optimal follow-up of patients with gallbladder cancer who are treated with curative intention. Routine imaging studies and endoscopic examination are not recommended and can be performed as clinically indicated. Follow-up investigations should be individualized based on the stage of the cancer, adjuvant treatment provided, performance status, and clinical signs and symptoms.

### 10.2. Locally Advanced Unresectable Gallbladder Cancer

The optimal management of patients with locally advanced and unresectable gallbladder cancer is controversial, and there is no internationally embraced standard approach. The options for patients with locally advanced gallbladder cancers include fluoropyrimidine chemoradiation or gemcitabine-based chemotherapy (such as gemcitabine/cisplatin combination) or fluoropyrimidine-based chemotherapy. The available data suggest that tumour control is rarely achieved with external beam radiation alone [101, 102]. Most patients with locally advanced unresectable disease are treated with combination of chemotherapy and radiation rather than radiation alone. However, it is not known if chemoradiation therapy is superior to chemotherapy alone in this setting and there is a lack of level 1 evidence validating this approach. There is limited evidence that chemoradiation therapy with or without surgery (trimodality therapy) in selected patients with locally advanced gallbladder cancers may result in prolonged survival [103]. If restaging in patients with locally advanced disease shows potentially resectable tumours (conversion therapy), resection should be considered. The NCCN clinical practice guidelines and the ESMO Guidelines Working Group in biliary cancer support concomitant fluoropyrimidine-based chemoradiotherapy as a treatment option to palliative chemotherapy for patients with locally advanced, unresectable gallbladder cancer [97, 104].

### 10.3. Metastatic Gallbladder Cancer

#### 10.3.1. Chemotherapy in Gallbladder Cancer

Systemic chemotherapy has shown significant but modest survival benefit in the management of advanced gallbladder cancer. A randomized trial compared systemic chemotherapy of gemcitabine plus oxaliplatin or 5-FU plus leucovorin versus best supportive care alone in 81 patients with unresectable gallbladder cancer [105]. Median overall survival in best supportive care and 5-FU/leucovorin groups was 4.5 and 4.6 months, respectively, versus 9.5 months in gemcitabine plus oxaliplatin group.

Of note, most published trials are small and have included patients with all biliary tract cancers. Only few clinical trials were performed exclusively in patients with gallbladder cancer [106–108]. There are three phase 2 trials that exclusively evaluated patients with gallbladder cancer. One study evaluated gemcitabine monotherapy and two trials assessed gemcitabine and cisplatin combination therapy (Table 5). In these trials, responses varied from 36 to 48% and median overall survival varied from 20 to 30 weeks. A pooled analysis of 104 chemotherapy trials involving 1,368 patients with biliary tract and gallbladder cancers that was conducted in 1985–2006 suggested differences in clinical behavior and responsiveness to chemotherapy between gallbladder and other biliary tract cancers. Pooled response rates and tumour control rates were 22.6 and 57.3%, respectively. Subgroup analysis showed superior response rate for gallbladder cancer compared with cholangiocarcinoma (36 versus 18%) but shorter overall survival for gallbladder cancer (7.2 versus 9.3 months) [109].

Most studies reported here are performed in patients with adenocarcinoma, the most common histology of gallbladder cancer. There is paucity of data regarding treatment of advanced adenosquamous or squamous cell gallbladder cancers, and in clinical practice these patients are treated similarly.

*(1) Fluoropyrimidine-Based Regimens*. 5-FU and 5-FU-based regimens were among the first reported in gallbladder cancers. In old trials, 5-FU alone or 5-FU-based combination therapies demonstrated objective response rates from 0 to 34% and median survival of four to six months in patients with advanced gallbladder and biliary tract cancers [110–112]. In contrast, most recent studies using infusional 5-FU combination therapy reported higher response rates and better overall survival [113–116]. In one study infusional 5-FU in combination with cisplatin resulted in partial response in six patients (24%). Median survival for patients with gallbladder cancer was 11.5 months [114].

Capecitabine is an orally active fluoropyrimidine derivative that has demonstrated efficacy in gallbladder cancer both as a single agent and in combination with cisplatin, gemcitabine, and oxaliplatin [117–121]. For instance, in a study involving 63 patients with hepatobiliary malignancies, which included eight patients with gallbladder cancer, capecitabine produced an objective response in four patients with gallbladder cancer, two of which were complete response [119]. In another trial involving 65 patients with biliary tract tumours, capecitabine was used in combination with oxaliplatin. Of 65 patients, 27 had gallbladder cancer. The patients with gallbladder cancer had a total disease control rate of 63% (one complete response, seven partial responses, and nine patients with stable disease) and a median survival of 8.2 months [120]. 

*(2) Gemcitabine-Based Regimens*. Gemcitabine is an active agent both as monotherapy and in combination regimens [107, 117, 118, 122, 123]. It has been extensively evaluated in patients with metastatic gallbladder and biliary tract cancer. The clinical benefit rates (partial response plus stable disease) with single agent gemcitabine are varied from 15 to 60% with overall response rates being as low as 7% [107, 122, 124–127]. Most studies reported median survival of 10 months or less. In contrast, reported response rates with gemcitabine combination therapies are varied from 17% to 50%, with median overall survival of up to 14 months (Table 6) [117, 118, 128–136]. At least four studies of gemcitabine plus cisplatin in patients with advanced gallbladder and biliary tract cancers have been reported. The reported response rates ranged from 21% to 34.5% and median survival times varied from 9.3 to 11 months [128–131]. The substitution of carboplatin for cisplatin decreases the severity of nonhematologic toxicity such as nausea, vomiting, nephropathy, and neuropathy; however, myelosuppression is sometimes worse. In a small trial, combination of gemcitabine and carboplatin was associated with response rate of 37% and median overall survival of about 11 months [137]. Several trials have demonstrated efficacy and good tolerability with a combination of gemcitabine and oxaliplatin [132–134]. The Groupe Coopérateur Multidisciplinaire en Oncologie study evaluated 56 patients with gallbladder and biliary tract cancers [133]. These patients were treated with gemcitabine and oxaliplatin combination and were stratified based on Eastern Cooperative Oncology Group performance status score (0–2 versus >2) and bilirubin. The median overall survival of patients with good performance status was almost double that of patients with poor performance status (15.4 months versus 7.6 months). Of note, even patients with poor performance status tolerated this regimen fairly well. Others report a far lower objective response rate with this regimen in advanced gallbladder cancer (1 of 23 patients, 4%) as compared to nongallbladder biliary tract carcinomas (9 of 44, 21%). Similar to gemcitabine and platinum compounds combination, gemcitabine and the oral 5-FU prodrug capecitabine combination has been associated with higher response rates than gemcitabine plus 5-FU for advanced biliary and gallbladder tumours. At least four phase II trials report response rates up to 32% and a median survival of approximately 13 to 14 months [117, 118, 135, 136].

The result of a randomized phase III trial that reported improvement in outcomes of patients with locally advanced or metastatic biliary tract and gallbladder cancers who were treated with combination therapy was a major breakthrough in management of advanced gallbladder and biliary tract cancers [126]. In this trial, 410 patients with locally advanced (25%) or metastatic bile duct (*n* = 242), gallbladder, (*n* = 149) or ampullary (*n* = 20) cancer were randomly assigned to eight courses of cisplatin (25 mg/m^2^) followed by gemcitabine (1000 mg/m^2^) on days 1 and 8, every 21 days, or gemcitabine alone (1000 mg/m^2^ on days 1, 8, and 15, every 28 days). At a median follow-up of 8.2 months, median progression-free survival (8 versus 5 months) and median overall survival (11.7 versus 8.1 months) were better with combination therapy.

*(3) Taxanes and Other Chemotherapeutic Agents*. Other chemotherapeutic agents have demonstrated limited benefit in gallbladder and biliary tract cancers. For instance, when paclitaxel was given every 21 days it demonstrated minimal efficacy in gallbladder cancer [138]. Likewise, the addition of pemetrexed to fixed-dose-rate gemcitabine, in a biweekly schedule, did not enhance the activity of gemcitabine in patients with biliary tract or gallbladder carcinoma [139]. Whereas docetaxel has shown a response rate of 20% in patients with advanced gallbladder and biliary tract cancers [140], single agent irinotecan demonstrated partial response rate of 8% and clinical benefit rate (partial response and stable disease) in 48% [141]. Based on the trial by Valle et al. [126] for patients with metastatic gallbladder cancer and good performance status, combination of cisplatin and gemcitabine is standard first line systemic therapy. In patients with borderline performance status, single agent gemcitabine or capecitabine is a reasonable alternative option.

#### 10.3.2. Second-Line Therapy in Gallbladder Cancer

Currently there is no “standard” second-line therapy after failure of first-line gemcitabine and cisplatin in patients with gallbladder cancer. In a preliminary report of 18 patients with advanced gemcitabine-refractory pancreaticobiliary cancer who received CAPOX, one had a partial response, and 8 patients had stable disease with the median progression-free survival of about 16 weeks in all patients [142]. Several targeted therapies in combination with chemotherapy have shown modest clinical benefit (see below). In patients with good performance status oxaliplatin-based regimen, 5-FU/capecitabine, taxanes, or irinotecan based therapy may be considered following progression on cisplatin/gemcitabine.

#### 10.3.3. Targeted Therapies in Gallbladder Cancer

Common mutations reported in gallbladder cancer are KRAS (10%–67%), EGFR (63%), BRAF (0% to 33%), and erbB2/HER2 (16%–64%) [23, 143, 144]. Early data suggest possible benefit from blockade of the epidermal growth factor receptor by the oral tyrosine kinase inhibitor erlotinib or anti-EGFR monoclonal antibody cetuximab (Table 7). [145–159]. A phase III Korean trial evaluated the efficacy of first-line treatment with gemcitabine and oxaliplatin with or without erlotinib in patients with advanced biliary tract cancer that included 31% of patients with gallbladder cancer. The median progression-free survival (PFS) was 5.8 months in the chemotherapy plus erlotinib group compared with 4.2 months in the chemotherapy alone group (HR, 0.80; 95% CI, 0.61–1.03; *P* = 0.087). Median OS was 9.5 months for both groups. However, in a subgroup of patients with gallbladder cancer, no benefit of erlotinib was noted (HR, 0.9; 95% CI, 0.63 to 1.58) [159]. A randomized phase II study comparing gemcitabine plus oxaliplatin alone with the same chemotherapy regimen in combination with cetuximab demonstrated a higher 4-month PFS rate with the addition of cetuximab (44% versus 61%, resp.) [153].

Vascular endothelial growth factor (VEGF) is overexpressed in biliary tract cancers and has been proposed as a therapeutic target [145, 146, 151, 152, 156]. The efficacy of bevacizumab, a monoclonal antibody targeting VEGF, in combination with erlotinib was assessed in a phase II trial. Nine patients had partial response to double targeted therapy that was sustained beyond four weeks in six patients, with median response duration of 8.4 months. Overall stable disease was observed in about half of the treated patients [151]. Sunitinib and sorafenib have shown modest benefit in biliary tract and gallbladder cancers [145, 146]. Likewise, selumetinib, a BRAF inhibitor, triapine, a ribonucleotide reductase inhibitor, and imatinib, a tyrosine kinase inhibitor, have shown some efficacy in gallbladder and biliary tract cancers [150, 157, 158]. In contrast, lapatinib targeting erB2/HER2 and bortezomib, a proteasome inhibitor, failed to demonstrate benefit in gallbladder and biliary tract cancers [147–149].

## 11. Radiation

While GBC's propensity for locoregional spread and recurrence suggest it is a rational target for intraoperative and postoperative radiotherapy, the role of adjuvant radiotherapy is poorly described in the literature with conflicting and largely disappointing results obtained in a small number of patients. The combination of external beam radiotherapy with fluorouracil has shown encouraging results, but further investigation is required. External radiation may be considered in palliative patients; however, tumour radioresistance typically precludes it from achieving tumour control as an independent therapeutic modality. Transhepatic percutaneous intraluminal brachytherapy using Ir-192 has been used as a palliative therapy for obstructive jaundice due to bile duct obstruction [160].

## 12. Palliation

Many patients who present symptomatically have advanced disease in whom palliation is the primary goal of treatment. Palliative bypass surgery may alleviate some of the symptoms commonly associated with incurable gallbladder cancer including jaundice, pruritus, cholangitis, pain, and biliary tract/gastrointestinal obstruction.

Biliary obstruction may be relieved by a number of procedures including Roux-en-Y or jejunal loop anastomosis with common hepatic duct or left duct or segment II or Longmire bilioenteric anastomosis. Segment III cholangiojejunostomy remains the most popular, with an associated high morbidity approaching 50% and mortality of 3.17%. Common complications include anastomotic leak and wound infection. As radiological and endoscopic stenting continues to evolve and improve, the future role of palliative surgery remains undetermined. No distinct advantage has been shown for one approach versus the other; however, it is suggested that the quality of life may be improved when treated surgically as postoperatively patients do not have the tubes and stents that would be present when treated by interventional radiological techniques [8].

Obstruction is a potential complication of late stage gallbladder cancer. Gastric outlet obstruction occurs in up to 30% of patients with advanced gallbladder cancer and may benefit from palliative gastrojejunostomy. The mortality and morbidity rates are high at 7.2% and 42%, respectively [8]. Palliative patients with bowel obstruction may be candidates for intestinal bypass procedures; however, morbidity is high in those with extensive peritoneal disease.

## 13. Prognosis

Gallbladder cancer is generally considered to confer a poor prognosis as this tumour typically remains silent until an advanced and often noncurative stage. Historically gallbladder cancer had an overall 5-year survival less than 5%. The recent advent of aggressive surgical resection with advances in perioperative care has markedly improved outcomes [1]. Other studies, however, have shown no significant improvement in OS in the past 20 years [52]. A retrospective review of gallbladder cancer throughout the 20th century found the overall survival has increased from 3.6 months in 1915–1932 to 10 months at the beginning of the 21st century [48]. Ongoing improvements in surgical techniques have resulted in a decline of both morbidity and mortality. In patients who undergo R0 curative resection, 5-year survival, by contrast, is 21–69%. Nevertheless, the French Surgical Association has demonstrated that 85% of T3/T4 tumours have an overall survival of only 2–8 months [18].

A recent (2012) multivariant Cox proportional hazard survival model by Hari et al. identified independent predictors of disease-specific survival to include age, T1 subtype, tumour grade, tumour histology, radiation, and surgery type, while independent predictors of overall survival were age, T1 subtype, tumour grade, tumour histology, race, and surgical procedure [90]. Prognostic factors specific for stage III/IV disease include adjuvant chemotherapy, tumour differentiation, hepatic invasion, and surgical margin status [92]. A recent (2013) study by D'Hondt et al. showed that patients with incidentally discovered gallbladder cancer have a significantly greater curability rate compared with nonincidentally discovered gallbladder cancer [88].

### 13.1. Patient Factors

Patient age is a well-recognized predictive factor. On multivariate analysis, treatment at a younger age is predictive of improved disease-specific survival [90]. On clinical exam, jaundice is a negative predictive finding, as it commonly indicates obstruction distal to the common hepatic duct or proximal common bile duct, therefore indicating advanced disease. The presence of a palpable mass, indicative of advanced disease, similarly confers a worse prognosis.

### 13.2. Tumour Factors

T-stage is an important prognostic feature. While the overall 5-year survival for T2 tumours is 70%, it falls dramatically to 0% for T3 tumours. Similarly, the rate of distant metastases increases from 16% in T2 to 79% in T4 disease, and the risk of nodal involvement increases from 33 to 69% [7]. Incidentally discovered gallbladder cancer has a better prognosis compared with patients with preoperative suspicion likely because of an earlier stage at incidental discovery [161, 162].

Among pT2–4 tumours, two patterns of intramural invasion are described: infiltrative (infiltration into the muscle without muscle destruction) and destructive (infiltration and destruction of muscle layer). Destructive growth confers a significantly lower overall survival than infiltrative, with higher rates of lymphovascular invasion, nodal positivity, and scirrhous growth pattern [162].

The extent of nodal involvement is an important prognostic factor. Whereas both the location and number of nodes were significant on univariate analysis, only the number of positive nodes is significant on multivariate analysis [83]. Other studies have shown a marked improvement in 5-year survival between node-negative (58–77%) and node-positive (0–45%) diseases [47]. It has been suggested that involvement of the peripancreatic nodes is indicative of a worse outcome. In patients with positive nodes, regional lymph node dissection may improve patient survival provided R0 resection is feasible [92]. Five-year survival rates following cholecystectomy alone are 5% and 13% with cholecystectomy and liver resection [8]. In patients who present with advanced disease, median survival is 2–4 months [6]. In all stages of GBC, R0 resection is an independent positive prognostic factor [88].

Hepatic involvement is well recognized as an independent prognostic factor in several series. In fact, on cox proportional regression, the importance of liver involvement is so dominant that no other covariants remained significant. While many of the other prognostic factors described in this section have been found on univariate analysis to be significant, hepatic involvement is consistently significant on both univariate and multivariate analyses [88].

### 13.3. Histology

The histological subtype of gallbladder cancer is another important prognostic factor. Papillary carcinoma confers the best prognosis, whereas squamous and adenosquamous carcinomas are more aggressive with a poorer prognosis. Small cell carcinoma, though very rare, metastasizes early and death often occurs shortly after diagnosis. The presence of perineural invasion is additionally significant for a worse outcome [88].

### 13.4. Miscellaneous

(i) *Lysosomal protein transmembrane 4 beta allele* ^**∗**^ *2 (LAPTM4B)* is one of the two alleles of LAPTM4B, a cancer-related gene, that contains two 19-base pair sequences in the 5′ untranslated region of exon 1. It is associated with poor histopathological differentiation, higher TNM stage, and the presence of lymph node metastases, with a shorter overall and disease-free survival. This allele is present in 37.9% of gallbladder cancers compared with 24.8% of controls [163]. The LAPTM4B status is suggested to be used to preoperatively evaluate patients for operability [163, 164].
(ii) Expressions of* Nectin-2, DDX3, integrin-linked kinase*, and* peroxiredoxin-1* have been shown to be independent poor prognostic factors in squamous/adenosquamous carcinomas of the gallbladder; however, these have not been studied in the more common gallbladder adenocarcinomas [164].
(iii) The prognostic role of* mucin* (MUC) expression in gallbladder remains disputed with MUC1 and MUC4 overexpression correlating with progression in some studies but not in others [164].
(iv) Overexpression of* histone-lysine N-methyltransferase* EZH2 or loss of phosphatase and tensin homolog expression may be implicated in the carcinogenesis of gallbladder cancer and convey a poorer prognosis [164]. Similarly, p53, bcl-2, bax, and COX-2 are all implicated in the pathogenesis of gallbladder carcinomas [164, 165].
(v) In contrast,* CDX2* and hepatocyte antigen expression increases the overall survival [164].
(vi) Expression of the* L1 cell adhesion molecule* is reported at the invasive front of 63.8% of gallbladder carcinomas and is associated with high histologic grade, advanced pathologic T-stage, clinical stage, and positive lymphovascular invasion. L1 cell adhesion molecule is reported to be an independent risk factor for disease-free survival [166].
(vii) Overexpression of* Skp2*, a SKP1-CUL1-F-box protein, has been shown to confer a shorter overall survival [166].
(viii) Expressions of* N(neural)-cadherin and P(placental)-cadherin* are associated with increased tumour size, invasion, and lymph node metastases in both adenocarcinoma and squamous cell/adenosquamous carcinomas of the gallbladder and with higher TNM staging in adenocarcinomas. N-cadherin is expressed in 52% of squamous cell/adenosquamous carcinomas and 55% of adenocarcinomas of the gallbladder; P-cadherin is expressed in 50% and 52.5%, respectively. Expressions of both N-cadherin and P-cadherin are both independent poor prognostic factors on multivariate Cox regression analysis [35].
(ix) Overexpression of CD54 is identified in gallbladder carcinoma, particularly in advanced disease [166].
(x) Intratumoural FoxP3 (transcriptional factor forkhead P3) is involved in the development and function of regulatory T cells and has been reported to be elevated in gallbladder cancers. FoxP3 and IL-17 positivity is correlated with nodal metastases and TNM stage. Additionally, FoxP3 positivity is associated with a poor disease-free survival on multivariate analysis [167].
(xi) Epithelial cell adhesion molecule (EpCAM) overexpression is reported to predict a decreased survival; however, its expression is not correlated with tumour grade or disease stage. Approximately 60% of patients with gallbladder cancer express EpCAM [168].
(xii) Frizzled (FZD1) is a member of a family of transmembrane receptors to which Wnt genes bind; these genes are well recognized to play a key role in controlling proliferation, specification, polarity, and cell migration. Expression of FZD1 is significantly associated with a large tumour size, high TMN staging, and lymph node involvement, with increased propensity for invasion, and thereby it is associated with a decreased overall survival in patients with gallbladder squamous cell and adenocarcinomas [169].

*Prevention, Screening, and Future Directions*. Eradication of gallstones remains the ideal target for the prevention of gallbladder cancer given their well-described association with carcinogenesis and given the fact that they are easily detected by ultrasound examination with a prolonged lead time of 20 years [3]. In this context, the role of prophylactic cholecystectomy in asymptomatic patients remains poorly defined. Patients at high risk (stones >2-3 cm, associated polyps, nonfunctioning gallbladder, porcelain gallbladder, pancreaticobiliary reflux, segmental adenomyomatosis, and xanthogranulomatous cholecystitis) may significantly benefit from prophylactic cholecystectomy, particularly those in a high risk geographical location [3, 12]. It is also recommended that gastrectomy patients undergo a concomitant cholecystectomy as these patients are predisposed to delayed gastric emptying with increased incidence of gallstones and rarely gallbladder cancers [12]. Potentially, unnecessary patient morbidity and the increased cost however remain barriers to this practice.

The future therefore should be directed towards research to promote early accurate diagnosis and improve management strategies which is dependent on global collaboration between general surgeons, gastroenterologists, radiologists, pathologists, and molecular biologists. Proposed areas for such research may include improved understanding of the molecular carcinogenesis with subsequent innovation of targeted chemotherapeutics, higher detection rates on imaging at an early T-stage, and the development of consensus-based guidelines for the management of T1b tumours. The creation of a sensitive and specific screening modality is perhaps of utmost importance to promote early detection of GBC at a resectable, low T-stage.

## 14. Conclusions

Gallbladder cancer is uncommon with a high case fatality occurring over a wide geographical distribution. Risk factors include advanced age, female gender, cholelithiasis, porcelain gallbladder, gallbladder polyps, congenital biliary cysts, chronic infection, and smoking. Most gallbladder cancers, unfortunately, are discovered incidentally at routine cholecystectomy or present as advanced stage disease. The role of radiological imaging, therefore, is limited to the use of ultrasound, CT scans, and endoscopic/FNA procedures for diagnostic and staging purposes. Adenocarcinoma accounts for the majority of gallbladder cancers. Surgery is the only curative therapy for gallbladder cancer. However, at diagnosis, less than ~20% of patients are candidates for curative surgery. The extent of surgical intervention is dependent on the TNM stage of the disease and may range from simple cholecystectomy in T1a tumour to partial hepatectomy and regional lymph node dissection in ≥T2 tumours. This may require reexcision of the tumor bed following the definitive pathological report.

Regional nodal status and the depth of tumor invasion (T status) are the two most important prognostic factors. The role of adjuvant therapy in GBC is not well defined. Nevertheless, in ≥T2 or node-positive disease, due to the high risk of recurrence, six months of gemcitabine or fluoropyrimidine-based chemotherapy can be considered. Such systemic chemotherapy has shown modest survival benefit in the management of advanced gallbladder cancer and is recommended in patients with good performance status along with best supportive care.

Currently, targeted therapy has limited role in the management of gallbladder cancer. Thus, the status of gallbladder cancers over the last century has not shown any definitive improvement in overall survival and continues to be plagued by the presence of advanced disease at diagnosis. This is directly related to the continued lack of sensitive screening modalities for the detection of early disease. The future, therefore, for improved success in the management of this disease may have to be directed towards the development of sensitive and specific screening strategies with relevant improved molecular understanding of the underlying pathogenesis of this “orphan disease.”

## Figures and Tables

**Table 1:** Summary of the major genes implicated in gallbladder carcinogenesis as available in the published literature (2000–present).

	Gene	Expression in GBC	Tissues of comparison	Additional information	Reference(s)
Oncogene	KRAS	Higher (10–67%)	Adenoma (0%)	Marker of GBC in PBM No correlation with stage, histology, and survival	[23, 26, 29]
	EGFR	Higher (63.4%)	Dysplasia (71.4%) Hyperplasia (15.4%) Normal (0%)		[23]
	HER-2/neu (ERBB2)	Higher (16–64%)	Carcinoma in situ (0%) Gallstones (0%)	Marker of metastatic disease (70%) Marker of poor prognosis (10x mortality)	[23, 30, 31]

Tumor suppressor	TP53	Higher (58.3–100%)	Adenoma (10–20%) Normal (0%)	Unknown relation to prognosis More prominent with poor differentiation	[23, 32]
	P16	Lower (48.8%)	Adenoma (100%) Chronic cholecystitis (100%)	Related to poorer prognosis Negative correlation with cyclin D1	[33]
	Fragile histidine triad (FHIT)	Lower	Normal	Early change in carcinogenesis	[26]
	Retinoblastoma	Lower (58.5%)	Adenoma (100%) Cholecystitis (100%)	Causes cell proliferation, apoptosis, and developmental defects	[33]
	VHL	Lower (48.1%)	Peritumoral tissue (80.4%) Polyps (80%) Chronic cholecystitis (88.6%)	Marker progression, biological behavior, and prognosis	[34]

Adhesion molecules and mucins	Cadherins	Higher (N-cadherin 55%; P-cadherin 53%)	None	Associated with large tumor size, invasion, and node metastases	[35]
	MUC1	Higher (78%)	Normal tissue (absent)	Higher expression in more advanced tumours; poor survival	[36]
	Erythrocyte complement receptor 1 (CR1)	Lower	Chronic cholecystitis Cholelithiasis Normal	Role under investigation	[23]

Angiogenesis	Thrombospondin-1	Higher (74.5%)	Normal (0%) T1 cancer (0%)	Associated with venous involvement Predictor of vascular involvement and nodal metastases	[23]
	Cyclooxygenase-2	Higher (59.2–71.9%)	Normal (0–25%) Dysplasia (70.3%)	Associated with poor prognosis, mean survival, and tumor progression	[23, 26]
	VEGF-A	Higher (81%)	Chronic cholecystitis (5.1%)	Expression related to histologic grade, TNM stage, and prognosis	[37]

Cell cycle regulators	Cyclin E	Higher (33%)	Adenoma (12.5%)		[23]
	Cyclin D1	Higher (41–68.3%)	Adenoma (57.1–67%) Chronic cholecystitis (7.1%) Normal (0%)	Marker of lymphatic/venous involvement and lymph node metastases	[23, 33]
	P27Kip1	Lower (43–65%)	None		[23]

Apoptosis	Caspases	Higher (95%; caspase 3; 77%; caspases 6 and 8)	None	Higher extent apoptosis in grade II/III GBC compared with grade I/dysplasia	[23]
	Bcl-2	Higher (34.7%)			

**Table 2:** Nevin's staging.

Stage	Definition
I	Tumour invades mucosa
II	Tumour invades mucosa + muscularis
III	Tumour invades mucosa + muscularis + subserosa
IV	Tumour invades all 3 layers of gallbladder + cystic lymph node
V	Tumour extends into liver bed or metastases

**Table 3:** Japanese Biliary Surgical Society staging system.

Stage	I	II	III	IV
Capsular invasion	No capsular invasion (**S**_**0**_)	Suspected capsular invasion (**S**_**1**_)	Marked capsular invasion (**S**_**2**_)	Direct invasion of adjacent viscera (**S**_**3**_)

Hepatic invasion	No hepatic invasion (**Hinf**_**0**_)	Suspected hepatic invasion (**Hinf**_**1**_)	Marked hepatic invasion around gallbladder (**Hinf**_**2**_)	Extensive hepatic invasion (**Hinf**_**3**_)

Bile duct invasion	No involvement of extrahepatic bile duct (**Binf**_**0**_)	Suspected involvement of bile duct (**Binf**_**1**_)	Marked biliary involvement (**Binf**_**2**_)	Extensive involvement of bile duct (**Binf**_**3**_)

Lymph node metastases	No lymph node metastasis (**N**_**0**_)	Metastases to lymph nodes around extrahepatic bile duct (primary group, **N**_**1**_)	Metastases in lymph nodes of hepatoduodenal ligament (secondary group, **N**_**2**_) OR surrounding area (tertiary group, **N**_**3**_)	Metastases more distant than in stage III (fourth group, **N**_**4**_)

Liver metastasis	No liver metastases (**H**_**0**_)	No liver metastases (**H**_**0**_)	No liver metastases (**H**_**0**_)	Liver metastases in 1 lobe (**H**_**1**_) OR Small liver metastases in bilateral lobes (**H**_**2**_) OR Multiple liver metastases in bilateral lobes (**H**_**3**_)

Peritoneal dissemination	No peritoneal dissemination (**P**_**0**_)	No peritoneal dissemination (**P**_**0**_)	No peritoneal dissemination (**P**_**0**_)	Peritoneal dissemination near tumour (**P**_**1**_) OR Small number of peritoneal disseminations distant from tumour (**P**_**2**_) OR Multiple peritoneal disseminations distant from tumour (**P**_**3**_)

**Table 4:** TNM staging.

Stage	T-stage	N-stage	M-stage
0	T_is_	N_0_	M_0_
I	T_1_	N_0_	M_0_
II	T_2_	N_0_	M_0_
IIIA	T_3_	N_0_	M_0_
IIIB	T_1_, T_2_, T_3_	N_1_	M_0_
IVA	T_4_	N_0_, N_1_	M_0_
IVB	Any T	Any N	M_1_
	Any T	N_2_	M_0_

Primary Tumour (T): T_is_ Carcinoma in situ; T_1 _ Tumour invades lamina propria (a) or muscular layer (b); T_2_ Tumour invades perimuscular connective tissue; T_3_ Tumour perforates serosa and/or invades liver and/or other adjacent organs (stomach, duodenum, colon, pancreas, and extrahepatic bile ducts); T_4_ Tumour invades main porta vein or hepatic artery or multiple extrahepatic organs.

Regional Lymph Nodes (N): N_0_ No regional lymph node metastasis; N_1_ Metastases to nodes along cystic duct, common bile duct, hepatic artery, and/or portal vein; N_2_ Metastases to periaortic, pericaval, superior mesenteric artery, and/or celiac artery nodes.

Distant Metastasis (M): M_0_ No distant metastasis; M_1_ Distant metastasis.

**Table 5:** Results of three phase 2 trials which exclusively evaluated efficacy of chemotherapy in patients with advanced gall bladder cancer.

Regimen	Number of patients	Response rate (%)	Median overall survival
Gemcitabine monotherapy [107]	26	36	30 weeks
Gemcitabine and cisplatin [106]	30	37	20 weeks
Gemcitabine and cisplatin [108]	42	48	7 months

Modified from [2].

**Table 6:** Efficacy of gemcitabine combination therapy in patients with advanced gallbladder and biliary tract cancer.

Regimens	Number of patients	Response rate (%)	Median overall survival (months)
Gemcitabine and cisplatin			
Meyerhardt et al. [128]	33	21	9.7
Thongprasert et al. [129]	40	26	8.4
Lee et al. [130]	24	21	9.3
Kim et al. [131]	29	35	11
Gemcitabine and oxaliplatin			
Harder et al. [132]	31	26	11
André et al. [133]	33^*∗*^; 23^*∗∗*^	36^*∗*^; 22^*∗∗*^	15.4^*∗*^; 7.6^*∗∗*^
Gebbia et al. [134]	24	50	14
Gemcitabine an capecitabine			
Knox et al. [118]	45	31	14
Cho et al. [117]	44	32	14
Riechelmann et al. [135]	75	29	12.7
Iyer et al. [136]	12	17	14

^*∗*^Patients with good performance status; ^*∗∗*^patients with poor performance status. Modified from [2].

**Table 7:** Targeted therapy alone or in combination with chemotherapy in gallbladder and biliary tract cancer.

Targeted agent	Disease site	Number GBC/total	Line of therapy	Response rate	Comments
Single agent targeting VGF					
Sorafenib [145]	BTC	12/31	First	6%	
Sunitinib [146]	BTC	NA/56	Second	9%	
Single agent targeting HER2					
Lapatinib [147]	HCC or BTC	17 BTC/57	First & second	0% in BTC	
Lapatinib [148]	HCC or BTC	NA/9	Any	0%	Trial was stopped early due to futility
Other single agents					
Bortezomib [149]	BTC	6/20	First, second, third	5%	Trial was stopped early due to futility
Selumetinib [150]	BTC	7/28	Second	10%	
Doublet of targeted agents					
Bevacizumab + erlotinib [151]	BTC	10/53	First	17%	
Bevacizumab + erlotinib [152]	Upper GI cancer	16 BTC/102	Second or later	6%	
Targeted agents with chemotherapy					
GEMOX ± cetuximab [153]	BTC	NA/50	First	23%	Response rate in control group: 29%
GEMOX + cetuximab [154]	BTC	NA/30	First	63%	9 patients underwent resection after response
GEMOX ± cetuximab [155]	BTC	50/122	First	27.3%	Response rate in control group: 15%
GEMOX + bevacizumab [156]	BTC	NA/35	First & second	40%	
Gem + triapine [157]	BTC	18/33	First	9%	
5FU/LV + imatinib [158]	BTC	19/41	First	8%	
GEMOX + erlotinib [159]	BTC	82/268	First	30%	16% in chemotherapy arm alone. No difference in OS

BTC, biliary tract cancer; GEMOX, gemcitabine and oxaliplatin; HCC, hepatocellular cancer; NA, not applicable; OS, overall survival. Modified from [143].
